# Reticulate evolution: frequent introgressive hybridization among chinese hares (genus *lepus*) revealed by analyses of multiple mitochondrial and nuclear DNA loci

**DOI:** 10.1186/1471-2148-11-223

**Published:** 2011-07-28

**Authors:** Jiang Liu, Li Yu, Michael L Arnold, Chun-Hua Wu, Shi-Fang Wu, Xin Lu, Ya-Ping Zhang

**Affiliations:** 1 Laboratory for Conservation and Utilization of Bio-resource & Key Laboratory for Microbial Resources of the Ministry of Education, Yunnan University, Kunming, 650091, PR, China; 2 State Key Laboratory of Genetic Resources and Evolution, Kunming Institute of Zoology, Kunming 650223, China; 3 Department of Genetics, University of Georgia, Athens, Georgia 30602, USA; 4 Department of Zoology, College of Life Sciences, Wuhan University, Wuhan 430072, China; 5 Utah State University Department of Animal, Dairy & Veterinary Sciences Old Main Hill 4700 Center for Integrated Biosystems Rm315 Logan, UT 84322-4700, USA

## Abstract

**Background:**

Interspecific hybridization may lead to the introgression of genes and genomes across species barriers and contribute to a reticulate evolutionary pattern and thus taxonomic uncertainties. Since several previous studies have demonstrated that introgressive hybridization has occurred among some species within *Lepus*, therefore it is possible that introgressive hybridization events also occur among Chinese *Lepus *species and contribute to the current taxonomic confusion.

**Results:**

Data from four mtDNA genes, from 116 individuals, and one nuclear gene, from 119 individuals, provides the first evidence of frequent introgression events via historical and recent interspecific hybridizations among six Chinese *Lepus *species. Remarkably, the mtDNA of *L. mandshuricus *was completely replaced by mtDNA from *L. timidus *and *L. sinensis*. Analysis of the nuclear DNA sequence revealed a high proportion of heterozygous genotypes containing alleles from two divergent clades and that several haplotypes were shared among species, suggesting repeated and recent introgression. Furthermore, results from the present analyses suggest that Chinese hares belong to eight species.

**Conclusion:**

This study provides a framework for understanding the patterns of speciation and the taxonomy of this clade. The existence of morphological intermediates and atypical mitochondrial gene genealogies resulting from frequent hybridization events likely contribute to the current taxonomic confusion of Chinese hares. The present study also demonstrated that nuclear gene sequence could offer a powerful complementary data set with mtDNA in tracing a complete evolutionary history of recently diverged species.

## Background

The genus *Lepus *(the jackrabbit and hares; order Lagomorpha) is widespread in Africa, Europe, Asia, and North and Central America. The number of *Lepus *species currently recognized ranges from 24 to 32 [1-4]. In China, hares (*Lepus*) are distributed from the Qinghai-Tibetan Plateau to near sea level, and from the mainland to the island of Hainan. To date, the evolutionary history of Chinese hares (Lagomorpha: *Lepus*) is poorly defined, mainly due to uncertain taxonomic classification [2,3,5-7].

Many taxonomic schemes based on morphology have been proposed for Chinese hares and classifying them into various numbers of species. The earliest studies divided extant Chinese hares into seven species: *L. capensis *(Cape hare), *L. sinensis *(Chinese hare), *L. oiostolus *(Woolly hare), *L. mandshuricus *(Manchurian hare), *L. timidus *(Mountain hare), *L. yarkandensis *(Yarkand hare) and *L. hainanus *(Hainan hare) [8-12]. Subsequent analyses, incorporating a large number of morphometric features (e.g., body measurements and skull characters) conducted by Luo [13], assigned Chinese hares into nine species by adding 2 species: *L. melainus *(Manchurian black hare) and *L. comus *(Yunnan hare). Several recent classifications, however, have reignited the debate. Hoffmann & Smith [3] divided *L. capensis *into two species: *L*. *tibetanus *and *L. tolai*, and placed *L. melainus *into *L. mandshuricus*. Pan *et al*. [7] also did not support the specific status of *L. melainus*, and assigned *L. tibetanus *within *L. tolai *(corresponding to *L. capensis*). Wu *et al*. [14], based on four mitochondrial genes, also suggested that *L. mandshuricus *and *L. melainus *should be a single species, but rejected the specific status of *L. capensis*. Liu *et al*. [15], using skull characters and nuclear gene sequences, supported the synonymy of *L. melainus *and *L. mandshuricus*. Hence, taxonomic classifications within Chinese hares remain uncertain and necessitate further analyses that include additional taxa and discrete sequence data.

Hybridization among wild mammalian species may lead to the introgression of genes and genomes across the species barrier yielding a reticulate evolutionary pattern (evolution characterized by genetic exchange among different evolutionary lineages) among these species and thus taxonomic uncertainties. As reviewed by Nosil [16], speciation with gene flow could be common; however, the impact of hybridization on the speciation process in animals remains poorly defined. Studies have revealed that introgressive hybridization often leads to the complete replacement of mtDNA on a regional scale [17-22] or even throughout a species' distribution [23]. Such replacements appear to be more common than previously appreciated and can affect the origin and adaptation of organisms [24].

If hybridization occurs between recently diverged species, then morphological intermediates between these species may be produced thus contributing to the taxonomic confusion. Such a phenomenon seems to occur within the genus *Lepus*. Several previous studies have demonstrated that introgressive hybridization has occurred among some species within *Lepus*. Thulin *et al*. [25] and Thulin & Tegelström [26,27] demonstrated the unidirectional introgression of *L. timidus *mtDNA into *L. europaeus *that was introduced into Sweden during the 19th century. Morphological intermediates between the two species, believed to be hybrids, have also been observed since the introduction of *L. europaeus *[28]. In addition, hybrids between *L. timidus *females and *L. europaeus *males are easily acquired in captivity [29] and the F_1 _hybrids show intermediate characters [30]. Furthermore, Alves *et al*. [31] and Melo-Ferreira *et al*. [17,32] described an ancient mtDNA introgression of *L. timidus*, which is now extinct from Iberia, into *L. granatensis *and *L. europaeus *in the Iberian Peninsula. A reverse direction of mtDNA introgression from *L. europaeus *into *L. timidus *was also reported in the Alps by Zachos *et al*. [33] and in Russia by Thulin *et al*. [34]. n ear gene sequenceszation and evolution.

In view of these findings, it is possible that introgressive hybridization events may also occur between Chinese *Lepus *species, most of which overlap geographically with other species of this genus [3,7,13]. If introgression among *Lepus *species does indeed occur it may contribute to the current taxonomic confusion of Chinese hares [21,35].

The transfer of genes and genomes across the species barrier can provide novel genetic material for natural selection to work upon and, thus, may change the evolutionary direction of one or both of the intermixing taxa [36-46]. Previous studies, however, have revealed that there was a lack of nuclear DNA introgression in hares from Iberia and Sweden. Alves [47] used 14 autosomal protein loci to assay the genetic variability in *L. granatensis *populations from Iberia and detected no significant differentiation between the populations with introgressed *L. timidus *mtDNA and non-introgressed populations. Similar results were also obtained from a microsatellite analysis [48,49]. Based on SNP analyses of 10 autosomal, two X-linked and one Y-linked loci, Melo-Ferreira *et al*. [50] suggested that autosomal introgression appeared to be mostly sporadic or undetectable and that sex-chromosome introgression was completely absent in hare species of Iberia. Alves *et al*. [51] and Roca *et al*. [52] attributed the lack of nuclear introgression to recurrent crossing of hybrid females with males of the invading species, coupled with male hybrid sterility or inviability (Haldane's rule). This pattern of crossing could eventually lead to the replacement of one nuclear genome within a few generations. This inference was also supported by the results of Thulin *et al*. [53]. By analyzing microsatellites, Thulin *et al*. [53] detected a small number of specimens with genotypes consistent with those of F_1 _hybrids in Sweden. From this observation it was hypothesized that the transferred nuclear genes from *L. timidus *had disappeared from the *L. europaeus *populations during the 50 generations since the introduction and initial hybridization. In view of these reports, if introgression among Chinese *Lepus *species does indeed occur, what then is the genomic legacy of the introgressed nuclear genes and genomes? At one extreme, they may disappear over subsequent generations. At the other extreme, some hybrid genotypes in the Chinese populations may actually possess elevated fitnesses thus leading to continued introgression and the possibility of the fixation of introgressed nuclear DNA, and eventually to the formation of novel hybrid lineages (i.e. hybrid species).

In the present study, four mtDNA genes and one nuclear DNA locus from 124 Chinese hare individuals collected at 54 localities in China were analyzed in order to test for the possibility of introgressive hybridization and to help clarify the taxonomy of Chinese hares.

## Results

### MtDNA and Nuclear sequence characterizations

Sequences for four mtDNA genes, cytochrome b (Cyt b; 1140 bp), cytochrome c oxidase subunit I (COX I; 1450 bp), NADH dehydrogenase subunit 4 (ND4; 1378 bp) and control region (D-loop; 589 bp), were obtained successfully from 116 Chinese hare individuals, with eight museum skin samples failing to yield full-length sequences due to the poor quality of the isolated DNA. The mtDNA genes appear to be of mitochondrial origin, rather than nuclear copies of mtDNA-like pseudogenes, as indicated by the fact that the amino acid sequences of the Cyt b, COX I and ND4 genes did not possess premature stop codons or frameshifting insertions/deletions. In addition, the phylogenetic analyses based on separate mtDNA fragments were generally concordant (data not shown).

The sequence characteristics of the four mtDNA fragments and one nuclear gene, Stem cell factor (MGF), are summarized in Table 1. As seen from Table 1,

**Table 1:** Characteristics of the gene segments used in this study.

Gene	Total number of samples	Total number of sequences	Sequence length	Informative sites	Variable sites	Nucleotide Composition				Ti/Tv	Heterozygotes (%)
								
						A	T	G	C		
Cyt b	116	116	1140	262	297	28.2	30.3	12.7	28.8	6.6	
COI	116	116	1450	283	304	26.4	32.3	17.4	23.8	6.1	
ND4	116	116	1378	346	385	30.8	31.4	10.5	27.3	6.6	
D-loop	116	116	590	204	220	28.7	28.7	11.7	30.9	2.9	
Total mtDNA	116	116	4558	1095	1206	28.5	31.1	13.4	27.0	5.4	
MGF	119	201	593	44	58	29.7	32.9	17.2	20.1	2.5	70.59

a total of 201 sequences from 119 samples with a length of 593 base pairs were aligned at the MGF locus. Full-length sequences could not be obtained from five museum skin samples. A total of 72 haplotypes were defined from the 58 polymorphic sites, 44 of which were potentially phylogenetically informative.

### Phylogenetic inference from MtDNA

**Figure 1:**
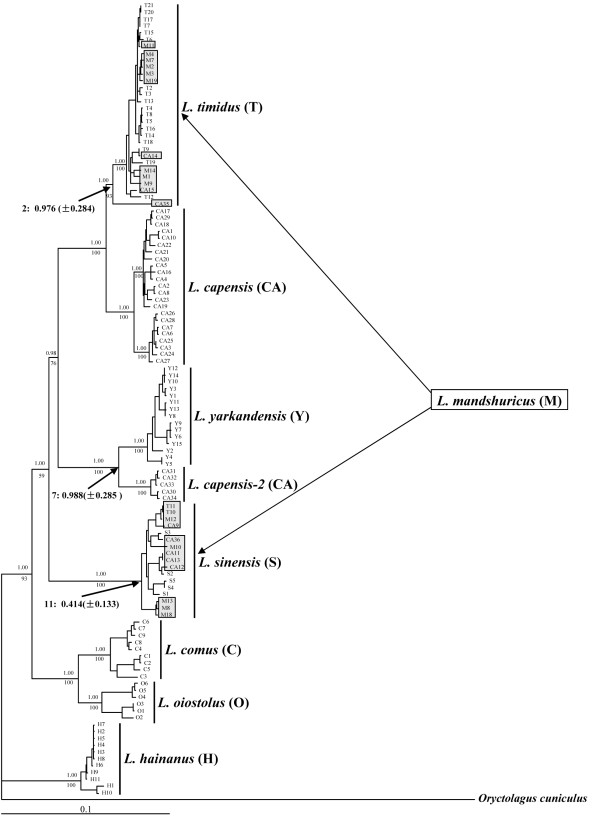
**The neighbour-joining (NJ) tree derived from the combined data of four mtDNA fragments**. *Oryctolagus cuniculus *was used as the outgroup. The numbers above the branches are the Bayesian posterior probabilities (PP) and the numbers below the branches are the bootstrap proportions (BS). Abbreviations of each species name are presented in the parentheses following the species name, and this abbreviation was used in the sequence names. Sequence names correspond to the sample codes listed in Additional file 4 and Additional file 2. The boxed and highlighted sequences indicated the samples with an introgressed mitochondrial genome. MtDNA of *L. mandshuricus *are completely replaced by mtDNA from *L. timidus *or *L. sinensis*, suggesting the absence of a "true" *L. mandshuricus *mtDNA lineage.

The best fitting model for the Bayesian analysis using the combined mtDNA data was "GTR+I+G" with the following parameter settings: Base = (0.2968, 0.2771, 0.1192, 0.3069), Nst = 6, Rmat = (3.2812, 75.0100, 6.1253, 2.5585, 57.7249, 1.0000), Rates = gamma Shape = 1.0115, and Pinvar = 0.5638. Neighbour-joining (NJ) and Bayesian analyses of the combined mtDNA dataset yielded identical tree topologies (Figure. 1). The Chinese *Lepus *species included eight lineages and were grouped into five major clades. Sequence divergence (uncorrected pairwise distance; p-distance) among the eight mtDNA lineages based on the separate mtDNA fragments are presented in Additional file 1 (2.2-8.5% in Cyt b, 2.6-8.6% in COX I, 3.2-11.1% in ND4 and 5.9-15.3% in the control region). As seen from Figure 1, *L. hainanus *branched first, followed by the sister-grouping of *L. comus *and *L. oiostolus*. In another portion of the phylogeny, *L. sinensis *was most basal, *L. timidus *and *L. capensis *are closely related, and *L. yarkandensis *and a portion of the *L. capensis *individuals, which are defined here as *L. capensis-2*, grouped together. The support for the monophyly of these eight lineages was 1.00 for Bayesian posterior probability (PP) and 93%-100% for NJ bootstrap sampling (BS). Support for relationships among the five clades was 0.98-1.00 for PP and 59%-93% for BS.

As illustrated in Figure 1, sequences from *L. hainanus*, *L. comus*, *L. oiostolus*, *L. sinensis *and *L. yarkandensis *showed species-specific monophyly;. however, this was not the case for *L. capensis*, *L. timidus *and *L. mandshuricus*. The sequences of *L. capensis *were mainly divided into two lineages. Besides the lineage that included the majority of *L. capensis *sequences, five sequences of *L. capensis *from Xinjiang Province of China formed a separate mtDNA clade (L.ca*pensis-2*) (1.00 for PP, 100% for BS), which was a sister clade to *L. yarkandensis*. In addition, another eight sequences of *L. capensis *were placed within two other lineages: three (CA14, CA15 and CA35) in a *L. timidus *lineage (1.00 for PP, 93% for BS), and five (CA9, CA11-CA13 and CA36) in a *L. sinensis *lineage (1.00 for PP, 100% for BS). Similarly, the sequences of *L. timidus *were also divided into two lineages: one included the majority of the *L. timidus *sequences, and the other included two sequences of *L. timidus *(T10 and T11) from Inner Mongolia Province of China that grouped within the *L. sinensis *lineage. Remarkably, we found that among the fourteen *L. mandshuricus *sequences, nine were placed within the *L. timidus *lineage and the remaining five grouped within the *L. sinensis *lineage.

### Phylogenetic inference from nuclear gene

**Figure 2:**
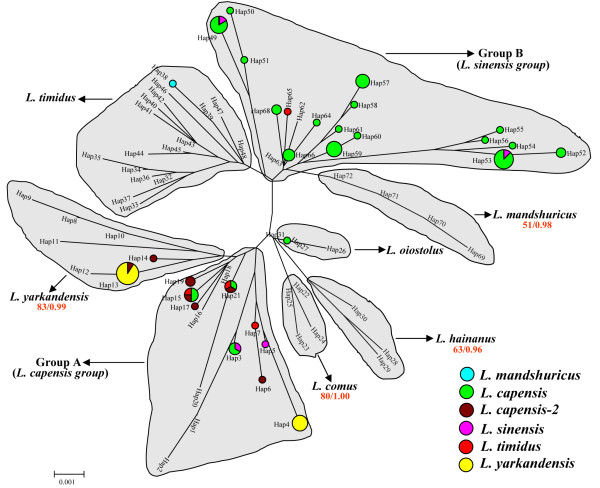
**The neighbour-joining (NJ) tree of 72 MGF haplotypes**. The numbers below the species names are the bootstrap proportions (BS) and the Bayesian posterior probabilities (PP) for the monophyly of that species. Only values of BS ≧ 50% or PP ≧ 0.50 are presented. Haplotypes that have been inferred to have been transfer across a species barrier or to be shared between species are highlighted with the colour pie charts. The relative sizes of the pie charts are proportional to the number of sequences within each haplotype. The evolutionary distances were computed using the maximum composite likelihood method and are in the units of the number of base substitutions per site.

The recombination test with the nuclear gene MGF gene data failed to detect any signal for recombination. The NJ tree for the nuclear gene MGF, based on the analyses of 72 haplotypes from the 119 individuals, is presented in Figure 2. The Bayesian inference (BI) analysis yielded a result similar to that of the NJ analysis.

The MGF gene tree in Figure 2 divided the Chinese *Lepus *species into six lineages (*L. hainanus*, *L. comus*, *L. oiostolus*, *L. yarkandensis*, *L. timidus *and *L. mandshuricus*) and two complexes (Group A and Group B). The haplotypes from *L. hainanus*, *L. comus, L. oiostolus*, *L. yarkandensis*, *L. timidus *and *L. mandshuricus *formed their own species-specific clades. Exceptions to this phylogenetic signal were one *L. capensis *haplotype (Hap31) that was included within the *L. oiostolus *clade, one *L. mandshuricus *haplotype (Hap38) that was included within the *L. timidus *clade, one *L. capensis-2 *haplotype (Hap14) that fell within the *L. yarkandensis *clade and one *L. yarkandensis *haplotype (Hap13) that was shared with *L. capensis-2*.

**Figure 3:**
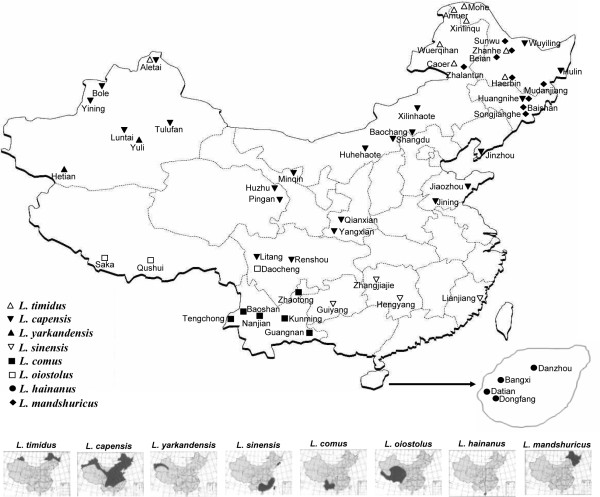
**Geographical distribution of the Chinese hare samples examined in this study**. The black ranges within the small figures at the bottom represent the distribution areas of each Chinese *Lepus *species.

The majority of the *L. sinensis *and *L. capeneis *haplotypes were admixed and fell within either Group A or Group B (Figure 2). Group A includes 14 haplotypes, of which five were from *L. capensis*, three (Hap6, 17, 19) were from *L. capensis-2*, and Hap 4, 5, 7 were from *L. yarkandensis*, *L. sinensis *and *L. timidus*, respectively. For the three remaining haplotypes, Hap15 and Hap21 were shared by *L. capensis*, *L. capensis-2 *and *L. timidus*, and Hap3 was shared by *L. capensis *and *L. sinensis*. Fourteen of the 19 haplotypes in Group B were from *L. capensis*. In addition, one *L. timidus *haplotype (Hap65), two *L. sinensis *haplotypes (Hap62, 63) and two haplotypes (Hap49, 53) shared between *L. sinensis *and *L. capeneis *were also grouped into Group B. One *L. yarkandensis *haplotype (Hap 4) was grouped into Group A and Hap13 was shared between *L. capensis-2 *and *L. yarkandensis*, suggesting a recent hybridization event between *L. yarkandensis *and a lineage in Group A. The disjunct geographical distributions of *L. yarkandensis *and *L. sinensis *(Figure 3) led to the definition of Group A as the "*L. capensis *group" and Group B as the "*L. sinensis *group" (Figure 2).

### Conflicts between the mitochondrial and nuclear gene trees

Three major differences were demonstrated between the mtDNA and nuclear gene trees: (i) no species-specific mtDNA lineage was identified for *L. mandshuricus*. All the mtDNA sequences of *L. mandshuricus *were identified as belonging to either *L. timidus *or *L. sinensis*; however, almost all of the MGF sequences from *L. mandshuricus *clustered together within a single clade; (ii) a new monophyletic lineage, *L. capensis-2*, was identified in the mtDNA analysis, but the MGF haplotypes of *L. capensis-2 *were divided into two portions (the *L. yarkandensis *clade and the *L. capensis *group); and (iii) the mtDNA analysis suggested that the introgression among Chinese *Lepus *species was likely unidirectional, while, in contrast, the MGF data indicated the occurrence of bidirectional introgression among these species. For example, from the mtDNA analysis, four *L. capensis *sequences were grouped with *L. sinensis*, suggesting the unidirectional introgression of mtDNA from *L. sinensis *into *L. capensis*; however, the MGF tree identified one haplotype (Hap3) which was nested in the *L. capensis *group and two haplotypes (Hap49, 53) which were nested in the *L. sinensis *group as sharing haplotypes between *L. capensis *and *L. sinensis*, consistent with the predicted pattern for bidirectional introgression. In addition, the fact that nine *L. capensis *and two *L. sinensis *individuals were identified as heterozygotes at the MGF locus, with alleles belonging to the *L. capensis *and *L. sinensis *groups, further supports the hypothesis of bidirectional introgression (Additional file 2). Details about the discrepancy between the mitochondrial and nuclear genes are summarized in Additional file 2.

### Dating the Divergence Times

The Bayesian tree used for divergence time estimation is presented in Additional file 3. In contrast to the mtDNA tree shown in Figure 1, the mtDNA tree in Additional file 3 included only representative sequences from each major lineage, and excluded sequences that demonstrated only a few base pair differences. The speciation time of Chinese hares was estimated at 3.116 MYA (± 0.731 MYA, Additional file 3). The estimated times of introgressive hybridization events are presented in Figure 1.

## Discussion

### Introgressive hybridization among Chinese Lepus species

As seen from the analyses of mtDNA and nuclear DNA sequences of Chinese hares, most of the phylogenetic conflicts between the mtDNA and nuclear DNA gene trees suggest multiple episodes of introgression. Alternatively, incomplete lineage sorting of the nuclear gene may also result in the observed "mixing" of the nuclear gene haplotypes; however, most of the samples with the discrepancy between the mitochondrial and nuclear genotypes have congruence between their morphology and nuclear genotype except for some *L. capensis *and *L. sinensis *individuals (Additional file 2). Although some of the *L. capensis *and *L. sinensis *individuals have incongruence between their morphology and nuclear genotype, the "*L. capensis *group" and the "*L. sinensis *group" is divergent enough to distinguish each other (Figure 2), therefore, the nuclear MGF gene is informative enough to distinguish Chinese hare species and the observed "mixing" of nuclear gene haplotypes likely is due to introgressive hybridization rather than incomplete lineage sorting. In addition, the observation that a large number of the heterozygous genotypes have alleles from divergent clades (Additional file 2) is also inconsistent with the alternative hypothesis of incomplete lineage sorting. The evidence suggests that introgression has occurred between seven pairs of the hare species examined here.

### L. sinensis × L. capensis, L. sinensis (♀) × L. mandshuricus (♂), L. sinensis × L. timidus

Five *L. capensis *individuals (CA9, CA11- CA13 and CA36) were placed within the *L. sinensis *lineage on the mtDNA tree (Figure 1). These results likely reflect recent introgression of *L. sinensis *mtDNA into *L. capensis*; however, results from the nuclear gene analysis suggested bidirectional and recent introgression between these species, as evidenced by the observation that the MGF haplotypes from *L. capensis *and *L. sinensis *were admixed and fell within two distantly related groups. Furthermore, one haplotype (Hap3) that nested in the *L. capensis *group and two haplotypes (Hap49, 53) that nested in the *L. sinensis *group were shared between *L. capensis *and *L. sinensis *(Figure 2). In addition, ten *L. capensis *individuals (CA1, CA2, CA10, CA17-CA19, CA21, CA22, CA29 and CA31) and two *L. sinensis *individuals (S2 and S3) were identified as heterozygotes at the MGF locus for two alleles from *L. capensis *and *L. sinensis *(Additional file 2). The available data, however, cannot eliminate the possibility that some of the heterozygotes were produced by ancient hybridization events.

Five individuals (M8, M10, M12, M13 and M18) of *L. mandshuricus *were grouped into the *L. sinensis *lineage on the mtDNA tree (Figure 1), while their MGF haplotypes fell within the *L. mandshuricus *clade (Figure 2). This can be explained by a unidirectional mtDNA introgression from *L. sinensis *into *L. mandshuricus*. In addition, two individuals of *L. mandshuricus *(M10 and M12) have low sequence divergence relative to the *L. sinensis *individuals, thus supporting a recent age for the hybridization events. The divergence time estimation indicated that the remaining three *L. mandshuricus *individuals (M8, M13 and MA18) shared a most recent ancestor with the *L. sinensis *samples at ca. 0.414 MYA (± 0.133 MYA) (Figure 1). This would suggest that the mitochondrial genome of *L. sinensis *introgressed into *L. mandshuricus *no later than 0.414MYA. Our estimate of the time of introgression may be biased somewhat if additional extant lineages were not sampled, or if there have been extinction events. Indeed, such a bias has been inferred in the case of *Lepidiolamprologus *[54]. However, in the present study three ancient hybridization events, involving five species (Figure 1; also see below), were detected. This strongly supports the hypothesis of ancient hybridization and not solely recent introgression events.

Two individuals of *L. timidus*, T10 and T11, nested within the *L. sinensis *clade on the mtDNA tree (Figure 1), while their MGF haplotypes grouped within the *L. timidus *clade (Figure 2), once again suggesting mtDNA introgression (in this case, from *L. sinensis *into *L. timidus*). In addition, the detection of one *L. timidus *haplotype (Hap65) within the *L. sinensis *group on the MGF gene tree (Figure 2) was consistent with the occurrence of nuclear DNA introgression from *L. timidus *into *L. sinensis*. Taken together, the mtDNA and nuclear DNA analyses supported reciprocal introgression between *L. timidus *and *L. sinensis*. An alternative explanation would be that the introgressive hybridization of *L. sinensis *× *L. capensis*, *L. sinensis *(♀) × *L. mandshuricus *(♂), and *L. sinensis *(♀) ×*L. timidus *(♂) resulted from an initial hybridization between *L. sinensis *and *L. capensis*, *L. mandshuricus *or *L. timidus*, followed by a second hybridization among *L. capensis*, *L. mandshuricus *and *L. timidus*. Supporting this latter hypothesis was the inference of hybridization between *L. timidus *and *L. mandshuricus *as well as *L. timidus *and *L. capensis *(see below).

### L. timidus (♀) × L. mandshuricus (♂), L. timidus (♀) × L. capensis (♂)

Alves *et al*. [51] suggested that mtDNA introgression from *L. timidus *might occur in several other regions in addition to Iberia and our results confirm this hypothesis. In the *L. timidus *mtDNA lineage, nine *L. mandshuricus *individuals (M1-M4, M7, M9, M11, M14 and M19) and three *L. capensis *individuals (CA14, CA15 and CA35) were included (Figure 1). The MGF haplotypes of the nine *L. mandshuricus *individuals were grouped with the other *L. mandshuricus *haplotypes except for Hap38, which is nested within the *L. timidus *clade. The nuclear sequences of the three *L. capensis *individuals were placed in either the *L. capensis *group (CA15) or the *L. sinensis *group (CA35) (Additional file 2). In addition, one *L. timidus *haplotype (Hap7) was placed into the *L. capensis *group and two haplotypes (Hap15 and Hap21) were shared with *L. capensis *on the MGF gene tree (Figure 2). Our results thus suggest that the *L. mandshuricus *and *L. capensis *individuals likely reflect the capture of *L. timidus *mitochondrial genomes via several hybridization events between females of *L. timidus *and males of *L. mandshuricus *or *L. capensis*. Furthermore, the nuclear DNA analysis indicated that the hybridization events between *L. timidus *and *L. mandshuricus *or *L. capensis *were recent and a second hybridization event likely occurred between *L. capensis *and *L. sinensis *as well. An additional nine Cyt b sequences of *L. mandshuricus *accessed from Genbank (accession numbers: AY650894-AY650899, AJ279423, DQ793162, DQ793163) were also grouped into the *L. timidus *lineage on our Cyt b tree (data not show), further supporting the inference of mtDNA introgression from *L. timidus *into *L. mandshuricus*. Finally, one *L. timidus *Cyt b sequence (accession number: AY745108) collected from GenBank was grouped within the *L. capensis *lineage in the Cyt b phylogeny (data not show), suggesting the introgression of mtDNA from *L. capensis *into *L. timidus*. The *L. capensis *individual, CA35, shared a most recent common ancestor with *L. timidus *samples at 0.976 MYA (± 0.284 MYA) (Figure 1), indicating that the hybridization between *L. timidus *and *L. capensis *took place at about this point in time.

### L. yarkandensis (♀) × L. capensis(♂), L. capensis × L. oiostolus

Several observations suggest the role of recent hybridization between *L. capensis *and *L. yarkandensis*. These observations include: one MGF haplotype (Hap4) of *L. yarkandensis *nested within the *L. capensis *group; one MGF haplotype (Hap13) of *L. yarkandensis *shared with *L. capensis-2 *(Figure 2); and two MGF alleles of four *L. yarkandensis *individuals (Y7, Y10, Y14, Y15) were grouped into either the *L. yarkandensis *and *L. capensis *groups (Additional file 2).

The newly identified *L. capensis-2 *lineage might have originated through ancient hybridization between female *L. yarkandensis *and male *L. capensis *at ca 0.988 MYA (± 0.285 MYA) (Figure 1; also see below). Interestingly, one *L. capensis *haplotype (Hap31) was grouped into the *L. oiostolus *clade (Figure 2). This may reflect a recent hybridization event between *L. capensis *and *L. oiostolus*. In addition, hybridization between *L. oiostolus *and *L. capensis *was also evidenced by the fact that one *L. oiostolus *Cyt b sequence (accession number: AJ279427) from GenBank was nested within the *L. capensis *lineage (data not shown).

### The possible forces driving the introgressive hybridization among Chinese Lepus species

Compared with introgression reported previously for other species complexes [18,21-23,54,55], especially within other *Lepus *species [17,25-27,31,32,50], Chinese hares demonstrated a previously unreported level of complexity. First, most of the previous studies within the genus *Lepus *reported only the presence of mtDNA introgression of *L. timidus*, in which unidirectional mtDNA introgression events from *L. timidus *into populations of *L. europaeus *in Sweden [25-27] and into *L. granatensis *and *L. europaeus *in Iberian Peninsula [17,31,32] were inferred, with the identification of nuclear introgression lacking or infrequent [48,50,53]. Similar scenarios have also been posited for several other species clades [18,22,23,54-56]. Second, although contemporaneous introgression has been reported for several non-hare species [21,57,58], most of these cases of introgression involve at the most two or three species. In comparison, the present study demonstrated that introgression among Chinese *Lepus *species has most likely been a continuous and recent process involving multiple waves of hybridization among six species.

Our results also suggest that introgression has occurred in multiple directions, involving both mitochondrial and nuclear DNA. The mtDNA of *L. mandshuricus *was replaced entirely by that of *L. timidus *and *L. sinensis*. Although several previous studies have provided convincing evidence for complete local or even range-wide replacement of mtDNA following hybridization in insects, fishes and reptilia [18,23,55,59], such observations are infrequent for mammals. The hybridization events among Chinese hares are possibly driven by species abundance asymmetries, mating preferences, range expansion and low divergence between *Lepus *species.

In the studies of Thulin & Tegelström [27], Hubbs [60] and Wirtz [61], they suggested that the females of a rare species (e.g. *L. timidus*) would generally hybridize with the males of a common species (e.g. *L. europaeus*). In the present study, the hybridization between females of *L. timidus *(rare species) and males of *L. capensis *(common species), and between females of *L. timidus *(rare species) and males of *L. mandshuricus *(common species) are congruent with their conclusion. However, the hybridization between females of *L. sinensis *(common species) and males of *L. capensis *(common species) is inconsistent with this hypothesis. In addition, Thulin & Tegelström [27] and Grant & Grant [62] indicated that unidirectional hybridization would usually occur between the females of smaller species (e.g. *L. timidus*) and the males of larger-bodied species (e.g. *L. europaeus*). Among Chinese hares, hybridization between females of *L. sinensis *(smaller species) and males of *L. capensis *(larger species), *L. timidus *(larger species) or *L. mandshuricus *(larger species) support this hypothesis. In contrast, the hybridizations between female *L. timidus *(larger species) and male *L. capensis *(smaller species) or *L. mandshuricus *(smaller species) are inconsistent with this hypothesis. Species abundance and body size, though, might have been different in ancestral populations. The present study indicates that at least some of the hybridization events are recent, therefore, the forces driving the hybridization among Chinese *Lepus *species may include species abundance asymmetries and mating preferences.

The hybridizations among Chinese *Lepus *species might be a consequence of range expansion caused by the Pleistocene glaciations. Melo-Ferreira *et al*. [32] estimated the timing of mtDNA introgression from *L. timidus *into *L. granatensis *in Iberia at 33 000-35 000 years and suggested that it might have been favored by the climatic fluctuation during the Pleistocene glacial phenomena. Our latest study also strongly indicates that one of the Chinese *Lepus *species (*Lepus yarkandensis*) has undergone repeated population reductions and expansions during the Pleistocene glacial and interglacial periods, respectively [63]. These population size fluctuations may have contributed to the extensive gene flow among *Lepus yarkandensis *populations. Similarly, the alternation of glacial and interglacial periods during the Pleistocene likely contributed to the conditions necessary for of the overlapping ranges of different Chinese hare species, resulting in the observed introgressive hybridization. Morgan *et al*. [59] and Melo-Ferreira *et al*. [32] drew similar inferences for both mosquitoes in Southeast Asia and European hares, respectively. Further support for the effect of glaciation comes from the estimation of some of the introgression events among the Chinese hare lineages as occurring during the Pleistocene (i.e. 0.414-0.988 MYA). Simulation studies have also suggested that range expansions could lead to asymmetric introgression from the resident species toward the invading species [64-66].

In addition to the above factors, the widespread and complex patterns of reticulate evolutionary events among the Chinese *Lepus *lineages may also reflect the generally low levels of interspecific genetic differentiation. Previous studies have suggested that *Lepus *experienced rapid radiation [67,68]. The lack of chromosomal structural changes supports such a model of relatively recent, and rapid, diversification in this clade [69,70]. Low levels of genetic diversification between species could result in relatively fertile F_1 _hybrids providing a bridge for further hybridization and thus introgression. Alternatively, low levels of genetic diversification between species may indicate that speciation processes of some Chinese *Lepus *species are not complete and there have been continuous gene flow among them. For example, the admixture of MGF gene between *L. sinensis *and *L. capensis *could be the consequence of an incomplete reproductive isolation between the two species. Further definition of the introgressive system characterizing Chinese *Lepus *species should provide a clearer resolution of the evolutionary factors that have resulted in this reticulate complex.

### Taxonomic classification of Chinese hares

Based on the mtDNA and nuclear gene sequence analyses, the present study not only suggests that introgressive hybridization among Chinese *Lepus *species contributed to the current taxonomic confusion, but also provides insights into the previously obscure taxonomic classification of Chinese hares. Our mtDNA and nuclear DNA analyses supported the specific status of eight hare species, including *L. hainanus*, *L. comus*, *L. oiostolus*, *L. yarkandensis*, *L. timidus*, *L. mandshuricus*, *L. capensis *and *L. sinensis*. This finding is consistent with the morphological taxonomy of Pan *et al*. [7]. The division of *L. capensis *into two species (*L. tibetanus *and *L. tolai*), as suggested by Hoffmann & Smith [3], was not favored in the present study.

In the mtDNA analysis of *L. mandshuricus*, nine specimens were found to harbor *L. timidus *mtDNA haplotypes and three specimens contained *L. sinensis *mtDNA haplotypes, suggesting the absence of a diagnostic *L. mandshuricus *mtDNA lineage (Figure 1); however, the nuclear gene (MGF) analysis supported the monophyly of *L. mandshuricus*, which is consistent with its specific status (Figure 2). Furthermore, Liu *et al*. [15], using skull characters and nuclear gene sequences, also supported the specific status of *L. mandshuricus*, therefore, the discrepancy between the mtDNA and nuclear gene trees is most likely due to mtDNA introgression from *L. timidus *and *L. sinensis *into *L. mandshuricus*. This finding reflects the importance of including nuclear markers for systematic studies of hares [71-73].

On the mtDNA tree, *L. capensis *and *L. timidus *formed sister taxa and have the lowest Cyt b sequence divergence (2.2%) among the species analyzed in this study (Figure 1, Additional file 1). The Cyt b sequence divergence of 7.6% between *L. capensis *and *L. sinensis *(Additional file 1), and the presence of *L. capensis *group and *L. sinensis *group on nuclear gene tree (Figure 2) supported the specific status of *L. capensis *and *L. sinensis*. As we discussed above, our analyses led to the inference that *L. timidus *and *L. sinensis *mitochondrial genomes had introgressed into *L. capensis *and that frequent nuclear DNA introgression had occurred between *L. capensis *and *L. sinensis*. These observations are inconsistent with those of Wu *et al*. [14], who concluded that *L. capensis *either does not exist in China as a unique taxon or has been replaced by *L. timidus*. In contrast, our data suggest the occurrence of continuous introgression from *L. timidus *into *L. capensis*.

In the present study, our mtDNA tree identified a new mtDNA lineage that might represent a previously unidentified hare species from Xinjiang Province. This is evidenced by the fact that the newly identified mtDNA lineage (*L. capensis-2*) was closely related to *L. yarkandensis *on the mtDNA tree (Figure 1) and the Cyt b sequence divergence (2.5%) between *L. capensis-2 *and *L. yarkandensis *is higher than that (2.2%) found between *L. capensis *and *L. timidus *(Additional file 1). This finding is consistent with the inference of Wu *et al*. [14] that there might be two or more new hare species in this area of China; however, the MGF haplotypes of the *L. capensis-2 *samples were divided into two different groups: (i) two (Hap13 and Hap 14) were nested in the *L. yarkandensis *clade, with one (Hap13) shared with *L. yarkandensis*; (ii) five (Hap6, 15, 17, 19, 21) fell into the *L. capensis *group, with two (Hap15 and Hap21) shared with *L. capensis *(Figure 2). In addition, the individuals of *L. capensis-2 *and *L. capensis *have no significant morphological differences, therefore, the alternative hypothesis, that this newly identified mtDNA lineage may reflect ancient mtDNA introgression from *L. yarkandensis *into *L. capensis*, seems more likely.

## Conclusions

This study has revealed the first evidence of complex, ancient and recent introgressive hybridization among Chinese *Lepus *species thus providing a framework for understanding the patterns of speciation and the taxonomy of this clade. Frequent introgressive hybridization involving *L. timidus L. oiostolus*, *L. sinensis*, *L. yarkandensis*, *L. capensis *and *L. mandshuricus *were detected. Both unidirectional mtDNA introgression and bidirectional nuclear DNA introgression were inferred. In contrast to the previously reported introgression events within the genus *Lepus*, some of the mtDNA introgression events among Chinese *Lepus *species were estimated to be relatively ancient. We hypothesized that asymmetries in species abundance, mating preferences, range expansion and low levels of genetic divergence between *Lepus *species may have been causal for this reticulate evolutionary process. The existence of morphological intermediates and atypical mtDNA genealogies resulting from this reticulation might have contributed to the current taxonomic confusion among Chinese *Lepus *species. Our analyses supported the classification of Chinese *Lepus *into eight species. The present study also demonstrated that nuclear DNA could offer a powerful complement to the use of mtDNA genes for tracing the evolutionary history of recently diverged species. Future studies incorporating broader taxonomic sampling and unlinked genetic markers should provide additional resolution of the reticulate evolutionary patterns and systematic relationships among Chinese hares.

## Methods

### Specimen collection

To avoid the impact of seasonal effect on the pelage color variations, a total of 124 Chinese hares were collected in winter from 54 localities in China (Additional file 4 and Figure 3). The taxonomic status of some Chinese hare species reflects a longstanding debate [2,3,5-7]. We adopted mainly the classification scheme of Luo [13] for this study, but assigned *L. melainus *into *L. mandshuricus*, therefore, there are eight putative Chinese *Lepus *species in our samples. As described by Luo [13], *L. capensis *and *L. sinensis *have significant differentiation in tail length; *L. capensis *has the longest tail length (80-125 mm) among Chinese *Lepus *species while *L. sinensis *has the shortest (30-60 mm). The winter pelage of *L. mandshuricus *samples examined here was entirely black, except for the ventral surfaces; this is a unique character for *L. mandshuricus*. In contrast, only *L. timidus *has a white winter pelage making the identification of animals belonging to this species similarly straightforward. *L. yarkandensis *has a smaller body size than *L. capensis *as evidenced by their average body weight (*L. yarkandensis*: 1635 g; *L. capensis*: 1987 g). In addition, a large, black speckle on the dorsal surface of the tail allows the identification of *L. capensis *individuals. All of the samples examined in the present analysis were collected from their typical distributional areas (Figure 3), further supporting the assignment of samples to various species. Samples with unavailable or uncertain morphological information were excluded from the present study. All collection was performed following animal use protocols approved by the Kunming Institute of Zoology Animal Care Committee.

### DNA extraction and PCR amplification

Total genomic DNA was extracted using a modified phenol/chloroform method [74,75]. For hair and skin specimens, total genomic DNA was extracted with the Chelex-100 method [75-77]. We designed hare-specific primers from the published sequences of lagomorph species, and those that we sequenced to amplify four mtDNA fragments (Cyt b, COX I, ND4 and D-loop) from 124 individuals (Additional file 5). Due to its better phylogenetic performance demonstrated in the Leporidae study of Matthee *et al*. [78], one nuclear DNA fragment (MGF) was also amplified from 124 individuals using primers from Matthee *et al*. [78,79]. The primers are situated in the MGF exon regions, and the faster evolving intron sequence was amplified. Interestingly, high proportion of heterozygotes was found at the MGF locus.

Amplified PCR products were purified and sequenced in both directions with an ABI PRISM 3730 DNA sequencer. Homology of the acquired sequences was assessed by BLAST searching [80] of GenBank. Direct sequencing of the PCR products from the nuclear gene allowed the identification of heterozygous genotypes. Products from heterozygous individuals were cloned into the PMD18-T vector (Takara, China) and transformed into an ultracompetent *E.coli *cell line (Takara, China). Six clones per ligation reaction were sequenced in both directions to identify the heterozygous positions. Since the same DNA samples were used to amplify both the mtDNA and nuclear gene fragments, and the sequencing of mtDNA fragments did have any signal of admixture, thus we could rule out contamination as a cause of the heterozygosity at the nuclear locus. The mtDNA and nuclear gene sequences obtained in the present study have been deposited into GenBank with the following accession numbers: ND4 (HM232860 - HM232975); Cyt b (HM232976 - HM233091); COX I (HM233092 - HM233207); D-loop (HM233208 - HM233323) and MGF (HM233559 - HM233732).

### Sequence data analysis

Sequences were aligned using Clustal X1.81 [81] and refined by visual inspection. Prior to phylogenetic analysis of nuclear sequence recombination tests were conducted by using Sawyer's method [82] with the program GENECONV [83]. The default parameters were used except that the mismatch penalties varied from small (gscale = 1) to infinite (gscale = 0).

Phylogenetic analyses were conducted using the NJ method in MEGA 4 [84] and BI method as implemented in MrBayes v3.0b4 [85] for the separate and combined mtDNA datasets and the nuclear DNA dataset. In the NJ analysis, gaps or missing data were excluded. Nodal supports were assessed using BS with 1000 replicates. The evolutionary distances were computed using the maximum composite likelihood method. Prior to BI analysis, the program Modeltest 3.7 was used to identify the optimal model of DNA substitution [86]. The best-fit model was selected according to the Akaike information criterion (AIC) [87]. Bayesian analysis began with random starting trees and ran for 5 × 10^6 ^generations, with the Markov chains sampled every 100 generations. We checked the average standard deviation of split frequencies for parameter convergence in the BI analysis. In our BI analysis, the average standard deviation of split frequencies was close to 0.007 when the run ended. The first 1.25 × 10^6 ^generations were excluded as burn-in. The analysis was conducted twice to ensure that the Bayesian analyses were not trapped in local optima [88,89]. The remaining trees from both analyses were used to create a majority rule consensus tree where the percent of samples recovering the same clade represented the PP value of that clade.

### Molecular clock test and divergence time estimation

The hypothesis of a molecular clock was examined for the combined mtDNA data set using the relative-rate test [90] with the program PHYLTEST [91]. Rate constancy was rejected at the 5% level, so we employed the relaxed Bayesian methods to estimate divergence times [92,93]. First, in the PAMLv3.14 package [94], we used BASEML to create the maximum likelihood output files needed for the MULTIDIVERGENCE package developed by Thorne *et al*. [92] and Kishino *et al*. [95]. Next, we used PAML2MODELINF to write "model files" needed to estimate the variance-covariance matrix for the four mtDNA fragments independently. The variance-covariance matrix estimation was performed using ESTBRANCHES. Finally, the output files from ESTBRANCHES were employed in MULTIDIVTIME to estimate the prior and posterior distributions of the divergence dates. Markov chain Monte Carlo analyses involved an initial burn-in (250,000 cycles), after which the Markov chain was sampled every 100th cycle a total of 20,000 times. Multiple independent runs were performed for the same data and prior distributions, but with different starting points to ensure stationarity. Two calibration points were used for estimating divergence time: (i) a literature-based divergence time between *L. comus *and *L. oiostolus *(1.83 ± 0.669MYA) [14]; (ii) the earliest fossil record of *L. timidus *collected from the Middle Pleistocene (0.75MYA) [96,97].

## Authors' contributions

JL, LY, CHW and YPZ designed the study. JL carried out the experiment work. JL and LY performed the sequence analyses. JL, LY and YPZ wrote the manuscript. MLA contributed to the manuscript revision. SFW and XL helped collect and identify samples. All authors read and approved the final manuscript.

## Supplementary Material

Additional file 1 **Uncorrected pairwise distances (P-distance) among eight mtDNA lineages of Chinese hares based on the separate analyses of four mtDNA fragments**. Click here for file

Additional file 2 **Details about the discrepancy between the mitochondrial and nuclear gene trees**. Sample code corresponds to sequence name shown in Figure 1 and sample code in Additional file 4. Click here for file

Additional file 3 **Bayesian tree used for divergence time estimates and the divergence time for each major clades**. Phylogenetic analyses are rooted with *Oryctolagus cuniculus*. The best substitute model GTR+I+G was used for Bayesian inference. The numbers above the branches are the Bayesian posterior probabilities (PP). Click here for file

Additional file 4 **Samples collected in this study**. Sample code corresponds to sequence name shown in Figure 1 and sample code in Additional file 2. Click here for file

Additional file 5 **Primers used for mitochondrial fragments amplification and sequencing**. Click here for file

## References

[B1] Corbet GB Hill JE A World list of mammalian species 1980 London: British Museum (Natural History)

[B2] Flux JEC Angermann R Chapman JA, Flux JEC The hares and jackrabbits Rabbits, Hares and Pikas: Status Conservation Action Plan 1990 Switzerland, Gland: International Union for Conservation of Nature and Natural Resources 61 94

[B3] Hoffmann RS Smith AT Wilson DE, Reeder DM Order Lagomorpha Mammal Species of the World 2005 3 Baltimore: Johns Hopkins University Press 198 205

[B4] Alves PC Ferrand N Hackländer K Lagomorph Biology: Evolution, Ecology, and Conservation 2008 Berlin Heidelberg: Springer

[B5] Angermann R The taxonomy of Old World *Lepus* Acta Zool Fennica 1983 174 17 21

[B6] Corbet GB Relationships and origins of European lagomorphs Mammal Rev 1986 16 105 110 10.1111/j.1365-2907.1986.tb00029.x

[B7] Pan QH Wang YX Yan K A Field Guide to the Mammals of China 2007 Beijing: China Forestry Publishing House

[B8] Allen GM The Mammals of China and Mongolia 1938 10 Pt. 1 Natural History of Central Asia, New York: American Museum of Natural History

[B9] Loukashkin AS On the Hares of Northern Manchuria J Mammal 1943 24 75 81

[B10] Tate GHH Mammals of Eastern Asia 1947 New York: MacMillan

[B11] Ellerman JR Morrison-Scott TCS Check list of Palaearctic and Indian mammals 1951 London: British Museum (Natural History)

[B12] Chen JSH The Fauna of Vertebrata in Taiwan 1956 Taiwan: Taiwan Commerce Publishing Company

[B13] Luo ZX The Chinese hare 1988 Beijing: China Forestry Publishing House

[B14] Wu CH Wu JP Bunch TD Li QW Wang YX Zhang Y-P Molecular phylogenetics and biogeography of *Lepus *in Eastern Asia based on mitochondrial DNA sequences Mol Phylogenet Evol 2005 37 45 61 10.1016/j.ympev.2005.05.006 15990340

[B15] Liu J Chen P Yu L Wu SF Zhang Y-P Jiang XL The taxonomic status of *Lepus melainus *(Lagomorpha: Leporidae) based on nuclear DNA and morphological analyses Zootaxa 2011 in press

[B16] Nosil P Speciation with gene flow could be common Mol Ecol 2008 17 2103 2106 10.1111/j.1365-294X.2008.03715.x 18410295

[B17] Melo-Ferreira J Boursot P Suchentrunk F Ferrand N Alves PC Invasion from the cold past: extensive introgression of mountain hare (*Lepus timidus*) mitochondrial DNA into three other hare species in northern Iberia Mol Ecol 2005 14 2459 2464 10.1111/j.1365-294X.2005.02599.x 15969727

[B18] Nyingi D Agnése JJ Recent introgressive hybridization revealed by exclusive mtDNA transfer from *Oreochromis leucostictus *(Trewavas, 1933) to *Oreochromis niloticus *(Linnaeus, 1758) in Lake Baringo, Kenya J Fish Biol 2007 70 Supplement A 148 154

[B19] Good JM Hird S Reid N Demboski JR Steppan SJ Martin-Nims TR Sullivan J Ancient hybridization and mitochondrial capture between two species of chipmunks Mol Ecol 2008 17 1313 1327 10.1111/j.1365-294X.2007.03640.x 18302691

[B20] Chen W Bi K Fu JZ Frequent mitochondrial gene introgression among high elevation Tibetan megophryid frogs revealed by conflicting gene genealogies Mol Ecol 2009 18 2856 2876 10.1111/j.1365-294X.2009.04258.x 19500253

[B21] Koblmüller S Nord M Wayne RK Leonard JA Origin and status of the Great Lakes wolf Mol Ecol 2009 18 2313 2316 10.1111/j.1365-294X.2009.04176.x 19366404

[B22] Runck AM Matocq MD Cook JA Historic hybridization and persistence of a novel mito-nuclear combination in red-backed voles (genus Myodes) BMC Evol Biol 2009 9 114 10.1186/1471-2148-9-114 19460158 PMC2697987

[B23] Nevado B Koblmüller S Sturmbauer C Snoeks J Usnao-Alemany J Verheyen E Complete mitochondrial DNA replacement in a Lake Tanganyika cichlid fish Mol Ecol 2009 18 4240 4255 10.1111/j.1365-294X.2009.04348.x 19780975

[B24] Arnold ML Fogarty ND Reticulate evolution and marine organisms: the final frontier? Int J Mol Sci 2009 10 3836 3860 10.3390/ijms10093836 19865522 PMC2769149

[B25] Thulin CG Jaarola M Tegelström H The occurrence of mountain hare mitochondrial DNA in wild brown hares Mol Ecol 1997 6 463 467 10.1046/j.1365-294X.1997.t01-1-00199.x 9161014

[B26] Thulin CG Tegelström H High mtDNA haplotype diversity among introduced Swedish brown hares *Lepus europaeus* Acta Theriol 2001 46 375 384 10.1007/BF03192444

[B27] Thulin CG Tegelström H Biased geographical distribution of mitochondrial DNA that passed the species barrier from mountain hares to brown hares (genus *Lepus*): an effect of genetic incompatibility and mating behaviour? J Zool 2002 258 299 306 10.1017/S0952836902001425

[B28] Lönnberg E On hybrids between *Lepus timidus *L. and *Lepus europeus *Pall. from southern Sweden Proc Zool Soc Lond 1905 1 278 287

[B29] Gustavsson I Sundt CO Anwendung von künstlicher befruchtung bei der hybridisierung von zwei Hasenarten Z Jagdwiss 1965 11 155 158 10.1007/BF01964745

[B30] Notini G Om harens biologi Sven Jägareförbundets Medd 1941 4 1 19218502912

[B31] Alves PC Ferrand N Suchentrunk F Harris DJ Ancient introgression of *Lepus timidus *mtDNA into *L. granatensis *and *L. europaeus *in the Iberian Peninsula Mol Phylogenet Evol 2003 27 70 80 10.1016/S1055-7903(02)00417-7 12679072

[B32] Melo-Ferreira J Boursot P Randi E Kryukov A Suchentrunk F Ferrand N Alves P C The rise and fall of the mountain hare (*Lepus timidus*) during Pleistocene glaciations: expansion and retreat with hybridization in the Iberian Peninsula Mol Ecol 2007 16 605 618 17257116 10.1111/j.1365-294X.2006.03166.x

[B33] Zachos FE Ben Slimen H Hackländer K Giacometti M Suchentrunk F Regional genetic *in situ *differentiation despite phylogenetic heterogeneity in Alpine mountain hares J Zool 2010 282 47 53 10.1111/j.1469-7998.2010.00710.x

[B34] Thulin CG Fang M Averianov AO Introgression from *Lepus europaeus *to *L. timidus *in Russia revealed by mitochondrial single nucleotide polymorphisms and nuclear microsatellites Hereditas 2006 143 68 76 10.1111/j.2006.0018-0661.01952.x 17362337

[B35] Koblmüller S Nord M Wayne RK Leonard JA More is better Mol Ecol 2009 18 4994 4996 10.1111/j.1365-294X.2009.04432.x 19366404

[B36] Arnold ML Natural hybridization and evolution 1997 Oxford: Oxford University Press

[B37] Grant PR Grant BR Hybridization of bird species Science 1992 256 193 197 10.1126/science.256.5054.193 17744718

[B38] Seehausen O van Alphen JJM Witte F Cichlid fish diversity threatened by eutrophication that curbs sexual selection Science 1997 277 1808 1811 10.1126/science.277.5333.1808

[B39] Andersson M Hybridization and skua phylogeny P Roy Soc Lond B Bio 1999 266 1579 1585 10.1098/rspb.1999.0818

[B40] Doiron S Bernatchez L Blier PU A comparative mitogenomic analysis of the potential adaptive value of arctic Charr mtDNA introgression in Brook Charr Populations (*Salvelinus fontinalis *Mitchill) Mol Biol Evol 2002 11 1902 1909 10.1093/oxfordjournals.molbev.a004014 12411599

[B41] Payseur BA Krenz JG Nachman MW Differential patterns of introgression across the × chromosome in a hybrid zone between two species of house mice Evolution 2004 58 2064 2078 15521462 10.1111/j.0014-3820.2004.tb00490.x

[B42] Seehausen O Hybridization and adaptive radiation Trends Ecol Evol 2004 19 198 207 10.1016/j.tree.2004.01.003 16701254

[B43] Martin NH Bouck AC Arnold ML Detecting adaptive trait introgression between *Iris fulva *and *I. brevicaulis *in highly selective field conditions Genetics 2006 172 2481 2489 16415358 10.1534/genetics.105.053538 PMC1456367

[B44] Teeter KC Payseur BA Harris LW Bakewell MA Thibodeau LM O'Brien J E Krenz JG Sans-Fuentes MA Nachman MW Tucker PK Genome-wide patterns of gene flow across a house mouse hybrid zone Genome Res 2008 18 67 76 18025268 10.1101/gr.6757907 PMC2134771

[B45] Arnold ML Martin NH Adaptation by introgression J Biol 2009 8 82 10.1186/jbiol176 19833002 PMC2776903

[B46] Fitzpatrick BM Johnson JR Kump DK Shaffer HB Smith JJ Voss SR Rapid fixation of non-native alleles revealed by genome-wide SNP analysis of hybrid Tiger Salamanders BMC Evol Biol 2009 9 176 10.1186/1471-2148-9-176 19630983 PMC2724412

[B47] Alves PC Caracterização genética e biologia reprodutiva da lebre Ibérica, Lepus granatensis. Análise filogenética, diferenciação populacional e ciclo anual de reprodução 2002 PhD thesis, University of Porto

[B48] Estonba A Solis A Iriondo M Sanz-Martin MJ Perez-Suarez G Markov G Palacios F The genetic distinctiveness of the three Iberian hare species: Lepus europaeus, *L. granatensis*, and *L. castroviejoi* Mamm Biol 2006 71 52 59

[B49] Freitas H Natural hybridization between the Iberian hare (Lepus granatensis) and the brown hare (L. europaeus) in northern Iberian Peninsula 2006 MSc thesis, Faculdade de Ciências do Porto, Porto, Portugal

[B50] Melo-Ferreira J Alves PC Freitas H Ferrand N Boursot P The genomic legacy from the extinct *Lepus timidus *to the three hare species of Iberia: contrast between mtDNA, sex chromosomes and autosomes Mol Ecol 2009 18 2643 2658 10.1111/j.1365-294X.2009.04221.x 19457181

[B51] Alves PC Melo-Ferreira J Freitas H Boursot P The ubiquitous mountain hare mitochondria: multiple introgressive hybridization in hares, genus *Lepus* Philos T R Soc B 2008 363 2831 2839 10.1098/rstb.2008.0053 PMC2606744 18508749

[B52] Roca AL Georgiadis N O'Brien SJ Cytonuclear genomic dissociation in African elephant species Nat Genet 2005 37 96 100 15592471 10.1038/ng1485

[B53] Thulin CG Stone J Tegelstrǒm H Walker CW Species assignment and hybrid identification among Scandinavian hares *Lepus europaeus *and *L. timidus* Wildlife Biol 2006 12 29 38 10.2981/0909-6396(2006)12[29:SAAHIA]2.0.CO;2

[B54] Schelly R Salzburger W Koblmüller S Duftner N Sturmbauer C Phylogenetic relationships of the lamprologine cichlid genus *Lepidiolamprologus *(Teleostei: Perciformes) based on mitochondrial and nuclear sequences, suggesting introgressive hybridization Mol Phylogenet Evol 2006 38 426 438 10.1016/j.ympev.2005.04.023 15964213

[B55] Renoult JP Geniez P Bacquet P Benoit L Crochet PA Morphology and nuclear markers reveal extensive mitochondrial introgressions in the Iberian Wall Lizard species complex Mol Ecol 2009 18 4298 4315 10.1111/j.1365-294X.2009.04351.x 19754512

[B56] Koblmüller S Duftner N Sefc KM Aibara M Stipacek M Blanc M Egger B Sturmbauer C Reticulate phylogeny of gastropod-shell-breeding cichlids from Lake Tanganyika-the result of repeated introgressive hybridization BMC Evol Biol 2007 7 7 10.1186/1471-2148-7-7 17254340 PMC1790888

[B57] Sota T Ishikawa R Ujiie M Kusumoto F Vogler AP Extensive trans-species mitochondrial polymorphisms in the carabid beetles *Carabus *subgenus *Ohomopterus *caused by repeated introgressive hybridization Mol Ecol 2001 10 2833 2847 11903896 10.1046/j.1365-294x.2001.t01-1-01404.x

[B58] Thompson SL Lamothe M Meirmans PG Périnet P Isabel N Repeated unidirectional introgression towards *Populus balsamifera *in contact zones of exotic and native poplars Mol Ecol 2010 19 132 145 20002578 10.1111/j.1365-294X.2009.04442.x

[B59] Morgan K Linton YM Somboon P Saikia P Dev V Socheat D Walton C Inter-specific gene flow dynamics during the Pleistocene-dated speciation of forest-dependent mosquitoes in Southeast Asia Mol Ecol 2010 19 2269 2285 10.1111/j.1365-294X.2010.04635.x 20444081

[B60] Hubbs CL Hybridization between fish species in nature Syst Zool 1955 4 1 20 10.2307/2411933

[B61] Wirtz P Mother species-father species: unidirectional hybridization in animals with female choice Anim Behav 1999 58 1 12 10.1006/anbe.1999.1144 10413535

[B62] Grant PR Grant BR Hybridization, sexual imprinting and mate choice Am Nat 1997 150 1 28 10.1086/286054 18811273

[B63] Shan WJ Liu J Yu L Murphy RW Halik M Zhang YP Genetic consequences of postglacial colonization by the endemic Yarkand hare (*Lepus yarkandensis*) of the arid Tarim Basin Chinese Sci Bull 2011 56 1370 1382 10.1007/s11434-011-4460-9

[B64] Currat M Excoffier L Modern humans did not admix with neanderthals during their range expansion into Europe PLoS Biol 2004 2 2264 2274 10.1371/journal.pbio.0020421 PMC532389 15562317

[B65] Currat M Ruedi M Petit RJ Excoffier L The hidden side of invasions: massive introgression by local genes Evolution 2008 62 1908 1920 18452573 10.1111/j.1558-5646.2008.00413.x

[B66] Excoffier L Foll M Petit RJ Genetic consequences of range expansions Annu Rev Ecol Evol S 2009 40 481 501 10.1146/annurev.ecolsys.39.110707.173414

[B67] Halanych KM Demboski JR Van Vuuren BJ Klein DR Cook JA Cytb phylogeny of North American hares and jackrabbits (*Lepus*, Lagomorpha) and the effects of saturation in outgroup taxa Mol Phylogenet Evol 1999 11 213 221 10.1006/mpev.1998.0581 10191066

[B68] Halanych KM Robinson TJ Multiple substitutions affect the phylogenetic utility of cytb and 12S rDNA data: examining a rapid radiation in leporid (Lagomorpha) evolution J Mol Evol 1999 48 369 379 10.1007/PL00006481 10093227

[B69] Robinson TJ Elder FFB Chapman JA Karyotype conservatism in the genus *Lepus *(order Lagomorpha) Can J Genet Cytol 1983 25 540 544 6652568 10.1139/g83-081

[B70] Azzaroli Puccetti ML Corti M Scanzani A Civitelli MV Cappana E Karyotypes of two endemic species of hare from Ethiopia, *Lepus habessinicus *and *L. starcki *(Lagomorpha, Leporidae). A comparison with *L. europaeus* Mammalia 1996 60 223 230 10.1515/mamm.1996.60.2.223

[B71] Robinson TJ Matthee CA Phylogeny and evolutionary origins of the Leporidae: A review of cytogenetics, molecular analyses and a supermatrix analysis Mammal Rev 2005 35 231 247 10.1111/j.1365-2907.2005.00073.x

[B72] Alves PC Harris DJ Melo-Ferreira J Branco M Suchentrunk F Boursot P Ferrand N Hares on thin ice: introgression of mitochondrial DNA in hares and its implications for recent phylogenetic analyses Mol Phylogenet Evol 2006 40 640 641 10.1016/j.ympev.2006.02.016 16624594

[B73] Ben Slimen H Suchentrunk F Shahin AB Ben Ammar Elgaaied A Phylogenetic analysis of mtCR-1 sequences of Tunisian and Egyptian hares (*Lepus *sp. or spp., Lagomorpha) with different coat colours Mamm Biol 2007 72 224 239 10.1016/j.mambio.2006.03.002

[B74] Sambrook J Fritsch EF Maniatis T Molecular Cloning 1989 Cold Spring Harbor, New York: Cold Spring Harbor Press

[B75] Wu CH Li HP Wang YX Zhang YP Low genetic variation of the Yunnan Hare (*Lepus comus *Allen 1927) as revealed by mitochondrial Cytb gene sequences Biochem Genet 2000 38 149 155 11091905

[B76] Walsh PS Metzger DA Higuchi R Chelex-100 as a medium for simple extraction of DNA for PCR based typing from forensic material Biotechniques 1991 10 506 513 1867860

[B77] Su B Monda K Wang W Jiang X Wang Y Woodruff DS Zhang Y Xia WP, Zhang YZ Molecular phylogeny of Chinese concolor gibbons (Subgeneus *Nomascus*) using noninvasive DNA genotyping Primate Research and Conservation 1995 Beijing: China Forestry publishing House 481

[B78] Matthee CA Van Vuuren BJ Bell D Robinson TJ A molecular supermatrix of the rabbits and hares (Leporidae) allows for the identification of five intercontinental exchanges during the Miocene Syst Biol 2004 53 433 447 10.1080/10635150490445715 15503672

[B79] Matthee CA Burzlaff JD Taylor JF Davis SK Mining the mammalian genome for artiodactyl systematics Syst Biol 2001 50 367 390 12116581

[B80] Altschul SF Madden TL Schäffer AA Zhang J Zhang Z Miller W Lipman DJ Gapped BLAST and PSI-BLAST: a new generation of protein database search programs Nucleic Acids Res 1997 25 3389 3402 10.1093/nar/25.17.3389 9254694 PMC146917

[B81] Thompson JD Gibson TJ Plewniak F Mougin FJ Higgins DG The Clustal × Windows interface: flexible strategies for multiple sequence alignment aided by quality analysis tools Nucleic Acids Res 1997 25 4876 4882 10.1093/nar/25.24.4876 9396791 PMC147148

[B82] Sawyer S Statistical tests for detecting gene conversion Mol Biol Evol 1989 6 526 538 2677599 10.1093/oxfordjournals.molbev.a040567

[B83] Sawyer S GENECONV: Statistical tests for detecting gene conversion (version 1.81) 2000 Washington University, St. Lousis, MO, USA Computer program distributed by the author

[B84] Tamura K Dudley J Nei M Kumar S MEGA4: Molecular Evolutionary Genetics Analysis (MEGA) software version 4.0 Mol Biol Evol 2007 24 1596 1599 10.1093/molbev/msm092 17488738

[B85] Ronquist F Huelsenbeck JP MrBayes 3: Bayesian phylogenetic inference under mixed models Bioinformatics 2003 19 1572 1574 10.1093/bioinformatics/btg180 12912839

[B86] Posada D Crandall KA Modeltest: testing the model of DNA substitution Bioinformatics 1998 14 817 818 10.1093/bioinformatics/14.9.817 9918953

[B87] Akaike H A new look at the statistical model identification IEEE Trans Autom contr 1974 19 716 723 10.1109/TAC.1974.1100705

[B88] Huelsenbeck JP Bollback JP Empirical and hierarchical Bayesian estimation of ancestral states Syst Biol 2001 50 351 366 12116580

[B89] Leaché AD Reeder TW Molecular systematics of the Eastern Fence lizard (*Sceloporus undulatus*): a comparison of parsimony, likelihood, and bayesian approaches Syst Biol 2002 51 44 68 10.1080/106351502753475871 11943092

[B90] Takezaki N Razhetsky A Nei M Phylogenetic test of the molecular clock and linearized trees Mol Biol Evol 1995 12 823 833 7476128 10.1093/oxfordjournals.molbev.a040259

[B91] Kumar S Phyltest: A Program For Testing Phylogenetic Hypothesis. Version 2.0 1996 The Pennsylvania State University, University Park

[B92] Thorne JL Kishino H Painter IS Estimating the rate of evolution of the rate of molecular evolution Mol Biol Evol 1998 15 1647 1657 9866200 10.1093/oxfordjournals.molbev.a025892

[B93] Thorne JL Kishino H Divergence time and evolutionary rate estimation with multilocus data Syst Biol 2002 51 689 702 10.1080/10635150290102456 12396584

[B94] Yang Z PAML: A program package for phylogenetic analysis by maximum likelihood Comput Appl Biosci 1997 13 555 556 9367129 10.1093/bioinformatics/13.5.555

[B95] Kishino H Thorne JL Bruno WJ Performance of a divergence time estimation method under a probabilistic model of rate evolution Mol Biol Evol 2001 18 352 361 11230536 10.1093/oxfordjournals.molbev.a003811

[B96] Kurtén B Pleistocene Mammals of Europe 1968 London: Weidenfeld and Nicholson

[B97] Kurtén B Anderson E Pleistocene Mammals of North America 1980 New York: Columbia University Press

