# Supplementary material for: Reticulate evolution: frequent introgressive hybridization among chinese hares (genus lepus) revealed by analyses of multiple mitochondrial and nuclear DNA loci
Source: BMC Evol Biol. 2011 Jul 28;11:223. doi: 10.1186/1471-2148-11-223 (PMC3155923; doi:10.1186/1471-2148-11-223)
Supplement: Additional file 1 — Uncorrected pairwise distances (P-distance) among eight mtDNA lineages of Chinese hares based on the separate analyses of four mtDNA fragments. [file 1471-2148-11-223-S1.DOC]

**Additional file 1** Uncorrected pairwise distances (P-distance) among eight mtDNA lineages of Chinese hares based on the separate analyses of four mtDNA fragments

| Taxa | 1 | 2 | 3 | 4 | 5 | 6 | 7 | 8 |
| --- | --- | --- | --- | --- | --- | --- | --- | --- |
| *L. hainanus* | **0.000**(COX I) |  |  |  |  |  |  |  |
|  | **0.002**(Cytb) |  |  |  |  |  |  |  |
|  | **0.006**(CR) |  |  |  |  |  |  |  |
|  | **0.013**(ND4) |  |  |  |  |  |  |  |
| *L. oiostolus* | 0.071 | **0.010** |  |  |  |  |  |  |
|  | 0.074 | **0.013** |  |  |  |  |  |  |
|  | 0.144 | **0.054** |  |  |  |  |  |  |
|  | 0.099 | **0.019** |  |  |  |  |  |  |
| *L. comus* | 0.071 | 0.032 | **0.011** |  |  |  |  |  |
|  | 0.079 | 0.039 | **0.012** |  |  |  |  |  |
|  | 0.129 | 0.104 | **0.045** |  |  |  |  |  |
|  | 0.096 | 0.048 | **0.011** |  |  |  |  |  |
| *L. sinensis* | 0.086 | 0.078 | 0.079 | **0.009** |  |  |  |  |
|  | 0.071 | 0.069 | 0.078 | **0.007** |  |  |  |  |
|  | 0.117 | 0.133 | 0.137 | **0.031** |  |  |  |  |
|  | 0.098 | 0.098 | 0.097 | **0.011** |  |  |  |  |
| *L. yarkandensis* | 0.08 | 0.075 | 0.077 | 0.078 | **0.004** |  |  |  |
|  | 0.081 | 0.058 | 0.065 | 0.073 | **0.007** |  |  |  |
|  | 0.125 | 0.131 | 0.129 | 0.128 | **0.027** |  |  |  |
|  | 0.11 | 0.111 | 0.102 | 0.099 | **0.007** |  |  |  |
| *L. capensis-2* | 0.077 | 0.067 | 0.066 | 0.078 | 0.031 | **0.002** |  |  |
|  | 0.083 | 0.06 | 0.059 | 0.067 | 0.025 | **0.003** |  |  |
|  | 0.132 | 0.144 | 0.153 | 0.14 | 0.059 | **0.012** |  |  |
|  | 0.11 | 0.105 | 0.101 | 0.102 | 0.042 | **0.003** |  |  |
| *L. timidus* | 0.076 | 0.068 | 0.066 | 0.069 | 0.064 | 0.055 | **0.005** |  |
|  | 0.082 | 0.059 | 0.068 | 0.063 | 0.059 | 0.063 | **0.007** |  |
|  | 0.125 | 0.12 | 0.127 | 0.107 | 0.108 | 0.112 | **0.031** |  |
|  | 0.085 | 0.093 | 0.082 | 0.084 | 0.087 | 0.088 | **0.007** |  |
| *L. capensis* | 0.081 | 0.074 | 0.073 | 0.074 | 0.067 | 0.057 | 0.026 | **0.008** |
|  | 0.085 | 0.059 | 0.067 | 0.076 | 0.057 | 0.059 | 0.022 | **0.006** |
|  | 0.148 | 0.13 | 0.147 | 0.115 | 0.126 | 0.124 | 0.083 | **0.038** |
|  | 0.097 | 0.092 | 0.032 | 0.092 | 0.095 | 0.095 | 0.032 | **0.007** |

*Note*. The bold values on the diagonal indicate sequence divergences within lineages. Values on the first line are the sequence divergences for the COX I sequences (COX I), the second for Cytb sequences (Cytb), the third for control region sequences (CR), and the fourth for ND4 sequences (ND4).
