# Supplementary material for: Reticulate evolution: frequent introgressive hybridization among chinese hares (genus lepus) revealed by analyses of multiple mitochondrial and nuclear DNA loci
Source: BMC Evol Biol. 2011 Jul 28;11:223. doi: 10.1186/1471-2148-11-223 (PMC3155923; doi:10.1186/1471-2148-11-223)
Supplement: Additional file 2 — Details about the discrepancy between the mitochondrial and nuclear gene trees. Sample code corresponds to sequence name shown in Figure 1 and sample code in Additional file 4. [file 1471-2148-11-223-S2.DOC]

**Additional file 2** Details about the discrepancy between the mitochondrial and nuclear genes. Sample code corresponds to sequence name shown in Figure 1 and sample code in Additional file 4.

| **Sample code** | **mtDNA** | **Nuclear DNA (MGF)** |
| --- | --- | --- |
| CA30, CA32 | *L. capensis-2* | *L. capensis* |
| CA33 | *L. capensis-2* | *L. yarkandensis* |
| CA3, CA5, CA6, CA8, CA23, CA24, CA25, CA26, CA28 | *L. capensis* | *L. sinensis* |
| CA9, CA11, CA12, CA13 | *L. sinensis* | *L. sinensis* |
| T10, T11 | *L. sinensis* | *L. timidus* |
| M8, M10, M12, M13, M18 | *L. sinensis* | *L. mandshuricus* |
| CA35 | *L. timidus* | *L. sinensis* |
| M1, M2, M3, M4, M7, M9, M11, M14, M19 | *L. timidus* | *L. mandshuricus* |
| T9, CA15 | *L. timidus* | *L. capensis* |
| Y12 | *L. yarkandensis* | *L. capensis* |
| CA1, CA2, CA10, CA17, CA18, CA19, CA21, CA22, CA29 | *L. capensis* | *L. capensis* |
|  |  | *L. sinensis* |
| CA27 | *L. capensis* | *L. capensis* |
|  |  | *L. oiostolus* |
| CA31 | *L. capensis-2* | *L. capensis* |
|  |  | *L. sinensis* |
| CA34 | *L. capensis-2* | *L. capensis* |
|  |  | *L. yarkandensis* |
| T16 | *L. timidus* | *L. capensis* |
|  |  | *L. timidus* |
| T14 | *L. timidus* | *L. sinensis* |
|  |  | *L. timidus* |
| M3 | *L. timidus* | *L. timidus* |
|  |  | *L. mandshuricus* |
| S2,S3 | *L. sinensis* | *L. capensis* |
|  |  | *L. sinensis* |
| Y7, Y10, Y14, Y15 | *L. yarkandensis* | *L. capensis* |
|  |  | *L. yarkandensis* |
