# Supplementary material for: Reticulate evolution: frequent introgressive hybridization among chinese hares (genus lepus) revealed by analyses of multiple mitochondrial and nuclear DNA loci
Source: BMC Evol Biol. 2011 Jul 28;11:223. doi: 10.1186/1471-2148-11-223 (PMC3155923; doi:10.1186/1471-2148-11-223)
Supplement: Additional file 3 — Bayesian tree used for divergence time estimates and the divergence time for each major clades. Phylogenetic analyses are rooted with Oryctolagus cuniculus. The best substitute model GTR+I+G was used for Bayesian inference. The numbers above the branches are the Bayesian posterior probabilities (PP). [file 1471-2148-11-223-S3.PDF]

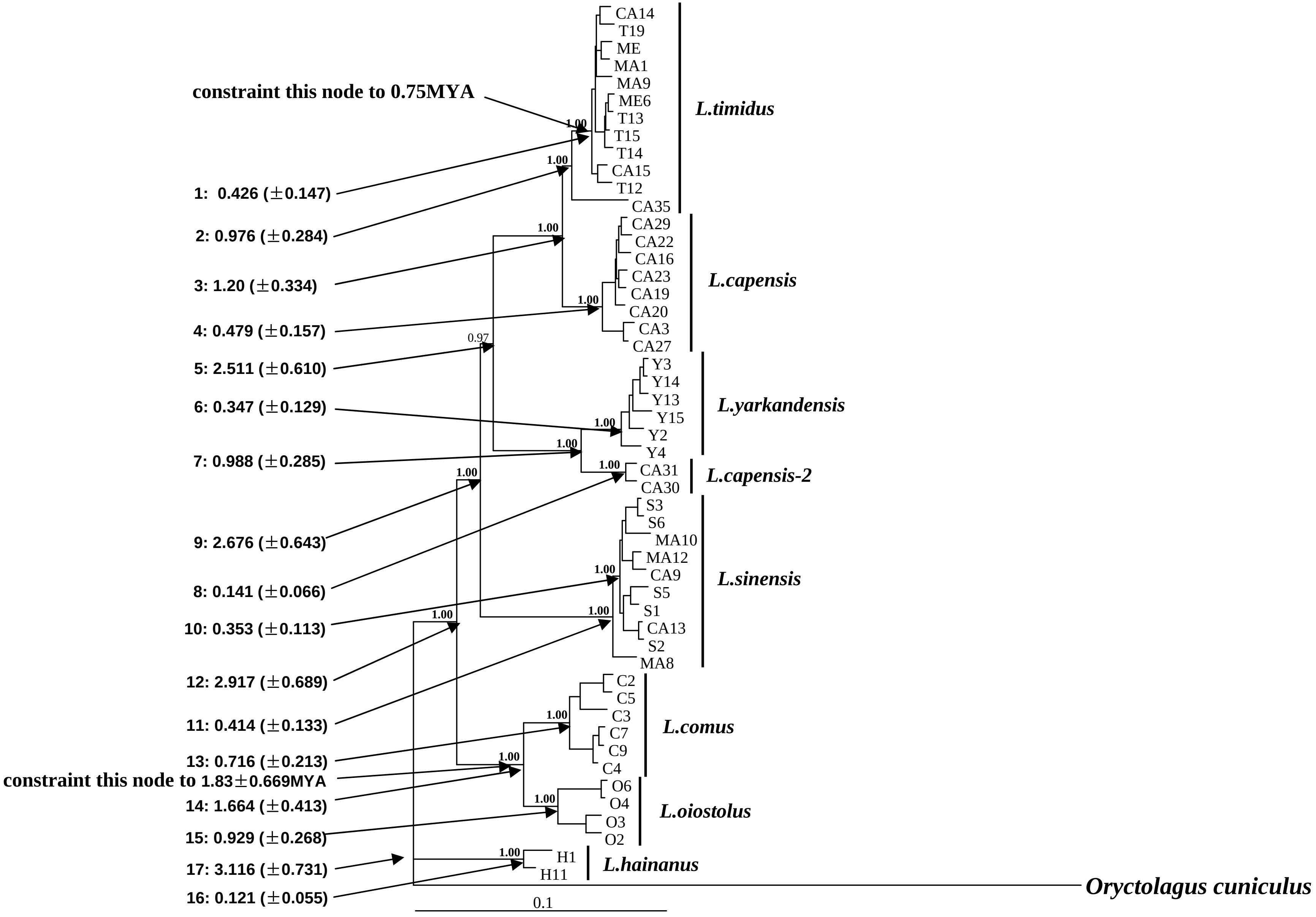

Additional file 3 Bayesian tree used for divergence time estimates and the divergence time for each major clades. Phylogenetic analyses are rooted with Oryctolagus *cuniculus*. The best substitute model GTR+I+G was used for Bayesian inference. The numbers above the branches are the Bayesian posterior probabilities (PP).
