# Supplementary material for: Reticulate evolution: frequent introgressive hybridization among chinese hares (genus lepus) revealed by analyses of multiple mitochondrial and nuclear DNA loci
Source: BMC Evol Biol. 2011 Jul 28;11:223. doi: 10.1186/1471-2148-11-223 (PMC3155923; doi:10.1186/1471-2148-11-223)
Supplement: Additional file 4 — Samples collected in this study. Sample code corresponds to sequence name shown in Figure 1 and sample code in Additional file 2. [file 1471-2148-11-223-S4.DOC]

**Additional file 4** Samples collected in this study. Sample code corresponds to sequence name shown in Figure 1 and sample code in Additional file 2

| Taxa | Sample code  codes | Collection locality |
| --- | --- | --- |
| *Lepus capensis* | CA1 | Minqin, Gansu Prov., China, N38°37′27.82″, E103°05′37.59″, elevation 1374 m |
|  | CA2 | Yangxian, Shanxi Prov., China, N33°13′22.27″, E107°32′43.84″, elevation 474 m |
|  | CA3 | Yangxian, Shanxi Prov., China, N33°13′22.27″, E107°32′43.84″, elevation 474 m |
|  | CA4 | Huzhu, Qinghai Prov., China, N36°50′39.91″, E101°57′28.65″, elevation 2536 m |
|  | CA5 | Huzhu, Qinghai Prov., China, N36°50′39.91″, E101°57′28.65″, elevation 2536 m |
|  | CA6 | Renshou, Sichuan Prov., China, N29°59′47.17″, E104°08′03.89″, elevation 482 m |
|  | CA7 | Renshou, Sichuan Prov., China, N29°59′47.17″, E104°08′03.89″, elevation 482 m |
|  | CA8 | Yangxian, Shanxi Prov., China, N33°13′22.27″, E107°32′43.84″, elevation 474 m |
|  | CA9 | Jinzhou, Liaoning Prov., China, N39°06′00.68″, E121°43′09.26″, elevation 27 m |
|  | CA10 | Minqin, Gansu Prov., China, N38°37′27.82″, E103°05′37.59″, elevation 1374 m |
|  | CA11 | Jiaozhou, Shandong Prov., China, N36°15′52.79″, E120°02′00.04″, elevation 39 m |
|  | CA12 | Jiaozhou, Shandong Prov., China, N36°15′52.79″, E120°02′00.04″, elevation 39 m |
|  | CA13 | Jiaozhou, Shandong Prov., China, N36°15′52.79″, E120°02′00.04″, elevation 39 m |
|  | CA14 | Yining, Xinjiang Prov., China, N43°54′46.60″, E81°19′45.57″, elevation 642 m |
|  | CA15 | Bole, Xinjiang Prov., China, N44°54′01.89″, E82°04′16.71″, elevation 563 m |
|  | CA16 | Pingan, Qinghai Prov., China, N36°30′02.03″, E102°06′31.81″, elevation 2140 m |
|  | CA17 | Hulin, Heilongjiang Prov., China, N45°45′37.14″, E132°56′10.65″, elevation 69 m |
|  | CA18 | Wuyiling, Heilongjiang Prov., China, N48°35′25.41″, E129°26′16.58″, elevation 414 m |
|  | CA19 | Xilinhaote, Neimenggu Prov., China, N43°56′00.28″, E116°05′09.72″, elevation 1039 m |
|  | CA20 | Baochang, Neimenggu Prov., China, N41°22′43.80″, E113°28′22.33″, elevation 1431 m |
|  | CA21 | Shangdu, Neimenggu Prov., China, N41°33′47.72″, E113°34′35.38″, elevation 1183 m |
|  | CA22 | Huhehaote, Neimenggu Prov., China, N40°50′32.32″, E111°44′55.85″, elevation 1072 m |
|  | CA23 | Pingan, Qinghai Prov., China, N36°30′02.03″, E102°06′31.81″, elevation 2140 m |
|  | CA24 | Qianxian, Shanxi Prov., China, N34°31′38.48″, E108°14′23.89″, elevation 647 m |
|  | CA25 | Jining, Shandong Prov., China, N35°24′53.51″, E116°35′13.47″, elevation 42 m |
|  | CA26 | Jining, Shandong Prov., China, N35°24′53.51″, E116°35′13.47″, elevation 42 m |
|  | CA27 | Jining, Shandong Prov., China, N35°24′53.51″, E116°35′13.47″, elevation 42 m |
|  | CA28 | Jining, Shandong Prov., China, N35°24′53.51″, E116°35′13.47″, elevation 42 m |
|  | CA29 | Huangnihe, Jilin Prov., China, N43°02′05.79″, E127°51′12.06″, elevation 739 m |
|  | CA30 | Bole, Xinjiang Prov., China, N44°54′01.89″, E82°04′16.71″, elevation 563 m |
|  | CA31 | Luntai, Xinjiang Prov., China, N41°46′40.05″, E84°15′06.96″, elevation 978 m |
|  | CA32 | Luntai, Xinjiang Prov., China, N41°46′40.05″, E84°15′06.96″, elevation 978 m |
|  | CA33 | Luntai, Xinjiang Prov., China, N41°46′40.05″, E84°15′06.96″, elevation 978 m |
|  | CA34 | Aletai, Xinjiang Prov., China, N47°50′41.73″, E88°08′28.51″, elevation 978 m |
|  | CA35 | Tulufan, Xinjiang Prov., China, N42°57′04.98″, E89°11′22.76″, elevation 170 m |
|  | CA36 | Litang, Sichuan Prov., China, N29°59′45.78″, E100°15′41.00″, elevation 4742 m |
| *L. timidus* | T2 | Haerbin, Heilongjiang Prov., China, N45°48′20.19″, E126°32′01.58″, elevation 116 m |
|  | T3 | Haerbin, Heilongjiang Prov., China, N45°48′20.19″, E126°32′01.58″, elevation 116 m |
|  | T4 | Amuer, Heilongjiang Prov., China, N52°52′39.32″, E123°39′58.90″, elevation 550 m |
|  | T5 | Amuer, Heilongjiang Prov., China, N52°52′39.32″, E123°39′58.90″, elevation 550 m |
|  | T6 | Caoer, Neimenggu Prov., China, N47°39′12.03″, E122°02′20.69″, elevation 623 m |
|  | T7 | Wuerqihan, Neimenggu Prov., China, N49°33′40.52″, E121°32′47.91″, elevation 792 m |
|  | T8 | Wuerqihan, Neimenggu Prov., China, N49°33′40.52″, E121°32′47.91″, elevation 792 m |
|  | T9 | Aletai, Xinjiang Prov., China, N47°50′41.73″, E88°08′28.51″, elevation 978 m |
|  | T10 | Caoer, Neimenggu Prov., China, N47°39′12.03″, E122°02′20.69″, elevation 623 m |
|  | T11 | Caoer, Neimenggu Prov., China, N47°39′12.03″, E122°02′20.69″, elevation 623 m |
|  | T12 | Mohe, Heilongjiang Prov., China, N52°58′05.47″, E122°32′37.25″, elevation 593 m |
|  | T13 | Mohe, Heilongjiang Prov., China, N52°58′05.47″, E122°32′37.25″, elevation 593 m |
|  | T14 | Mohe, Heilongjiang Prov., China, N52°58′05.47″, E122°32′37.25″, elevation 593 m |
|  | T15 | Mohe, Heilongjiang Prov., China, N52°58′05.47″, E122°32′37.25″, elevation 593 m |
|  | T16 | Xinlinqu, Heilongjiang Prov., China, N52°20′00.00″, E124°00′00.00″, elevation 615 m |
|  | T17 | Xinlinqu, Heilongjiang Prov., China, N52°20′00.00″, E124°00′00.00″, elevation 615 m |
|  | T18 | Xinlinqu, Heilongjiang Prov., China, N52°20′00.00″, E124°00′00.00″, elevation 615 m |
|  | T19 | Xinlinqu, Heilongjiang Prov., China, N52°20′00.00″, E124°00′00.00″, elevation 615 m |
|  | T20 | Zhanhe, Heilongjiang Prov., China, N48°13′43.37″, E126°41′46.76″, elevation 317 m |
|  | T21 | Zhanhe, Heilongjiang Prov., China, N48°13′43.37″, E126°41′46.76″, elevation 317 m |
| *L. yarkandensis* | Y1 | Yuli, Xinjiang Prov., China, N41°21′30.60″, E86°16′43.88″, elevation 914 m |
|  | Y2 | Yuli, Xinjiang Prov., China, N41°21′30.60″, E86°16′43.88″, elevation 914 m |
|  | Y3 | Yuli, Xinjiang Prov., China, N41°21′30.60″, E86°16′43.88″, elevation 914 m |
|  | Y4 | Hetian, Xinjiang Prov., China, N37°06′50.96″, E79°55′19.96″, elevation 1735 m |
|  | Y5 | Hetian, Xinjiang Prov., China, N37°06′50.96″, E79°55′19.96″, elevation 1735 m |
|  | Y6 | Hetian, Xinjiang Prov., China, N37°06′50.96″, E79°55′19.96″, elevation 1735 m |
|  | Y7 | Hetian, Xinjiang Prov., China, N37°06′50.96″, E79°55′19.96″, elevation 1735 m |
|  | Y8 | Hetian, Xinjiang Prov., China, N37°06′50.96″, E79°55′19.96″, elevation 1735 m |
|  | Y9 | Hetian, Xinjiang Prov., China, N37°06′50.96″, E79°55′19.96″, elevation 1735 m |
|  | Y10 | Hetian, Xinjiang Prov., China, N37°06′50.96″, E79°55′19.96″, elevation 1735 m |
|  | Y11 | Hetian, Xinjiang Prov., China, N37°06′50.96″, E79°55′19.96″, elevation 1735 m |
|  | Y12 | Hetian, Xinjiang Prov., China, N37°06′50.96″, E79°55′19.96″, elevation 1735 m |
|  | Y13 | Hetian, Xinjiang Prov., China, N37°06′50.96″, E79°55′19.96″, elevation 1735 m |
|  | Y14 | Hetian, Xinjiang Prov., China, N37°06′50.96″, E79°55′19.96″, elevation 1735 m |
|  | Y15 | Hetian, Xinjiang Prov., China, N37°06′50.96″, E79°55′19.96″, elevation 1735 m |
|  | Y16 | Yuli, Xinjiang Prov., China, N41°21′30.60″, E86°16′43.88″, elevation 914 m |
| *L. sinensis* | S1 | Lianjiang, Fujian Prov., China, N26°11′50.55″, E119°32′23.00″, elevation 12 m |
|  | S2 | Guiyang, Guizhou Prov., China, N26°38′45.35″, E106°38′00.15″, elevation 1290 m |
|  | S3 | Guiyang, Guizhou Prov., China, N26°38′45.35″, E106°38′00.15″, elevation 1290 m |
|  | S4 | Hengyang, Hunan Prov., China, N26°53′37.82″, E112°34′18.84″, elevation 78 m |
|  | S5 | Zhangjiajie, Hunan Prov., China, N29°06′58.81″, E110°28′44.80″, elevation 188 m |
| *L. oiostolus* | O1 | Daocheng, Sichuan Prov., China, N29°02′13.23″, E100°17′54.25″, elevation 4444 m |
|  | O2 | Daocheng, Sichuan Prov., China, N29°02′13.23″, E100°17′54.25″, elevation 4444 m |
|  | O3 | Saka, Tibet, China, N29°19′42.51″, E85°13′59.96″, elevation 4873 m |
|  | O4 | Qushui, Tibet, China, N29°21′11.18″, E90°44′37.77″, elevation 3593 m |
|  | O5 | Qushui, Tibet, China, N29°21′11.18″, E90°44′37.77″, elevation 3593 m |
|  | O6 | Qushui, Tibet, China, N29°21′11.18″, E90°44′37.77″, elevation 3593 m |
|  | O7 | Qushui, Tibet, China, N29°21′11.18″, E90°44′37.77″, elevation 3593 m |
| *L. comus* | C1 | Tengchong, Yunnan Prov., China, N25°01′14.22″, E98°29′24.01″, elevation 1634 m |
|  | C2 | Tengchong, Yunnan Prov., China, N25°01′14.22″, E98°29′24.01″, elevation 1634 m |
|  | C3 | Baoshan, Yunnan Prov., China, N25°06′43.43″, E99°09′41.30″, elevation 1677 m |
|  | C4 | Zhaotong, Yunnan Prov., China, N27°20′17.73″, E103°43′02.87″, elevation 1916 m |
|  | C5 | Nanjian, Yunnan Prov., China, N25°02′35.61″, E100°30′34.63″, elevation 1599 m |
|  | C6 | Guangnan, Yunnan Prov., China, N24°02′47.33″, E105°03′18.93″, elevation 1400 m |
|  | C7 | Kunming, Yunnan Prov., China, N25°02′14.22″, E102°43′19.65″, elevation 1900 m |
|  | C8 | Kunming, Yunnan Prov., China, N25°02′14.22″, E102°43′19.65″, elevation 1900 m |
|  | C9 | Kunming, Yunnan Prov., China, N25°02′14.22″, E102°43′19.65″, elevation 1900 m |
| *L. hainanus* | H1 | Dongfang, Hainan Prov., China, N19°05′43.30″, E108°39′06.39″, elevation 8 m |
|  | H2 | Datian, Hainan Prov., China, N20°29′20.54″, E109°34′21.54″, elevation -20 m |
|  | H3 | Datian, Hainan Prov., China, N20°29′20.54″, E109°34′21.54″, elevation -20 m |
|  | H4 | Datian, Hainan Prov., China, N20°29′20.54″, E109°34′21.54″, elevation -20 m |
|  | H5 | Datian, Hainan Prov., China, N20°29′20.54″, E109°34′21.54″, elevation -20 m |
|  | H6 | Datian, Hainan Prov., China, N20°29′20.54″, E109°34′21.54″, elevation -20 m |
|  | H7 | Bangxi, Hainan Prov., China, N19°23′11.33″, E109°05′57.16″, elevation 61 m |
|  | H8 | Bangxi, Hainan Prov., China, N19°23′11.33″, E109°05′57.16″, elevation 61 m |
|  | H9 | Danzhou, Hainan Prov., China, N19°31′16.08″, E109°34′50.92″, elevation 143 m |
|  | H10 | Danzhou, Hainan Prov., China, N19°31′16.08″, E109°34′50.92″, elevation 143 m |
|  | H11 | Danzhou, Hainan Prov., China, N19°31′16.08″, E109°34′50.92″, elevation 143 m |
|  | H12 | Dongfang, Hainan Prov., China, N19°05′43.30″, E108°39′06.39″, elevation 8 m |
| *L. mandshuricus* | M1 | Mudanjiang, Heilongjiang Prov., China, N44°35′15.48″, E129°36′52.79″, elevation 238 m |
|  | M2 | Sunwu, Heilongjiang Prov., China, N49°25′29.35″, E127°19′37.51″, elevation 220 m |
|  | M3 | Huangnihe, Jilin Prov., China, N43°02′05.79″, E127°51′12.06″, elevation 739 m |
|  | M4 | Zhalantun, Neimenggu Prov., China, N48°35′30.93″, E122°45′22.76″, elevation 615 m |
|  | M5 | Haerbin, Heilongjiang Prov., China, N45°48′20.19″, E126°32′01.58″, elevation 116 m |
|  | M6 | Haerbin, Heilongjiang Prov., China, N45°48′20.19″, E126°32′01.58″, elevation 116 m |
|  | M7 | Zhanhe, Heilongjiang Prov., China, N48°13′43.37″, E126°41′46.76″, elevation 317 m |
|  | M8 | Zhanhe, Heilongjiang Prov., China, N48°13′43.37″, E126°41′46.76″, elevation 317 m |
|  | M9 | Zhanhe, Heilongjiang Prov., China, N48°13′43.37″, E126°41′46.76″, elevation 317 m |
|  | M10 | Beian, Heilongjiang Prov., China, N48°14′06.97″, E126°29′57.42″, elevation 260 m |
|  | M11 | Zhanhe, Heilongjiang Prov., China, N48°13′43.37″, E126°41′46.76″, elevation 317 m |
|  | M12 | Songjianghe, Jilin Prov., China, N42°11′12.76″, E127°29′35.40″, elevation 704 m |
|  | M13 | Baishan, Jilin Prov., China, N41°56′54.40″, E126°25′53.32″, elevation 474 m |
|  | M14 | Haerbin, Heilongjiang Prov., China, N45°48′20.19″, E126°32′01.58″, elevation 116 m |
|  | M15 | Haerbin, Heilongjiang Prov., China, N45°48′20.19″, E126°32′01.58″, elevation 116 m |
|  | M16 | Haerbin, Heilongjiang Prov., China, N45°48′20.19″, E126°32′01.58″, elevation 116 m |
|  | M17 | Haerbin, Heilongjiang Prov., China, N45°48′20.19″, E126°32′01.58″, elevation 116 m |
|  | M18 | Zhanhe, Heilongjiang Prov., China, N48°13′43.37″, E126°41′46.76″, elevation 317 m |
|  | M19 | Zhanhe, Heilongjiang Prov., China, N48°13′43.37″, E126°41′46.76″, elevation 317 m |
