# Supplementary material for: Reticulate evolution: frequent introgressive hybridization among chinese hares (genus lepus) revealed by analyses of multiple mitochondrial and nuclear DNA loci
Source: BMC Evol Biol. 2011 Jul 28;11:223. doi: 10.1186/1471-2148-11-223 (PMC3155923; doi:10.1186/1471-2148-11-223)
Supplement: Additional file 5 — Primers used for mitochondrial fragments amplification and sequencing. [file 1471-2148-11-223-S5.DOC]

**Additional file 5** Primers used for mitochondrial fragments amplification and sequencing

| Sequence fragments | Primer name | Type | Sequences(5′to 3′) |
| --- | --- | --- | --- |
| COI | COIF1 | External | ATGTTCATCAATCGTTGA |
|  | COIR1 | External | GGTTCGATTCCTTCCTTTCTT |
|  | COIF470 | Internal | CACTTAGCTGGAGTTTCATCTA |
|  | COIR1050 | Internal | ATAGTAGTAATAAAATTAAT |
|  | COIF970 | Internal | CAGGGGTAAAAGTATTTAGTTG |
|  | COIR530 | Internal | TAAGAAAATAAAGCCTAAGGC |
| Cytb | CYTB80-F | External | CAACTACAAGAACCTAATGACCAA |
|  | CYTB1280-R | External | CAGGGTAATAYACTATACTACTGG |
|  | CYTB410-F | Internal | TGAGGMCAAATATCATTCTGAGG |
|  | CYTB370-R | Internal | AATGCTGTGGCTATTAC |
|  | CYTB730-F | Internal | ATACTCCTAGTCTTATTCTC |
| D-loop | D-LOOP 20-F | External | CAGAGATGGAGATYAACTCACC |
|  | D-LOOP 700-R | External | GCATGGGCTGATTAGTCATWAGT |
|  | D-LOOP400 | Internal | TGACTTGGATGGTSTATG |
| ND4 | ND4F1 | External | AATCTGAACCTTCTACAATGCTAA |
|  | ND4R1600 | External | AGCAGTTCTTGCAAGCATTCTC |
|  | ND4F480 | Internal | TAATGACATTCTCAGCCACAGA |
|  | ND4F850 | Internal | TCTATAGTCCTAGCAGCCAT |
|  | ND4R-970 | Internal | AGGTCTGTTTGTCGAAGGCAA |
|  | ND4430 | Internal | TCCTGCATTRAGTCGYTCTGT |
